# Feasibility of high-resolution DWI of the healthy dental pulp in human using a hybrid TGSE BLADE sequence

**DOI:** 10.1038/s41598-025-16272-2

**Published:** 2025-09-12

**Authors:** Mia A. Sittinger, Christoph Sandner, Kun Zhou, Johannes Mente, Arne Lauer, Ali C. Özen, Dominik F. Vollherbst, Michael O. Breckwoldt, Martin Bendszus, Sabine Heiland, Tim Hilgenfeld

**Affiliations:** 1 Department of Neuroradiology, Heidelberg University Hospital, Heidelberg, Germany; 2 Department of Conservative Dentistry, Heidelberg University Hospital, Heidelberg, Germany; 3 Siemens Shenzhen Magnetic Resonance Ltd, Shenzhen, China; 4 Division of Medical Physics, Department of Diagnostic and Interventional Radiology, Faculty of Medicine, University Medical Center Freiburg, University of Freiburg, Freiburg im Breisgau, Germany

**Keywords:** Magnetic resonance imaging, Diffusion magnetic resonance imaging, Tooth, Dental pulp, Dental MRI, Maxillofacial, Magnetic resonance imaging, Dental radiology, Diagnostic markers

## Abstract

Clinical evaluation of the human dental pulp is limited to imprecise, subjective methods, necessitating the development of an accurate and objective diagnostic parameter. Diffusion-weighted imaging (DWI) could address this gap; however, no in vivo data are available. To illustrate the feasibility of DWI imaging of the human pulp and to investigate physiological patterns. A turbo gradient spin echo (TGSE) BLADE diffusion MRI sequence was optimized for dental pulp imaging in three volunteers, defining an optimal b-value range and assessing intraindividual fluctuations at 3 T. Apparent diffusion coefficient (ADC) values were calculated in 18 participants using the 2-point and 7-point methods in 329 healthy teeth, analyzing data according to tooth and jaw type. Statistical analysis was performed using Kruskal-Wallis, Wilcoxon signed-rank-test, and one-way ANOVA. Mean pulp ADCs (± SD) for the 2-/7-point methods were 1.219 ± 0.340 / 1.242 ± 0.297 × 10^−9^ m^2^/s (*p* ≤ 0.0001), with intraindividual variations of 0.159 × 10^−9^ m^2^/s. Minor but significant differences were observed between tooth types for maxillary premolars vs. maxillary incisors/canines, mandibular canines, and maxillary incisors vs. molars (ADC difference range: 0.209–0.272 × 10^−9^ m^2^/s; p-values: 0.0017–0.0473). In vivo assessment of the dental pulp by DWI is feasible and reliable using the TGSE BLADE sequence. Reference ADC values were established, with no substantial differences between jaw and tooth types, indicating that the ADC is a stable intraindividual parameter here. These findings may substantially improve the diagnostic evaluation of pulp diseases to minimize over- and undertreatment.

## Introduction

Diseases of the dental pulp constitute a significant proportion of dental diseases^1,2^. For a correct diagnosis it is crucial to ascertain the vitality of the pulp and ensure accurate classification, both of which are essentially related to its vascularization^3^. Direct measurement of the pulp’s vascular supply is currently only feasible experimentally for selected tooth types through the application of Doppler flowmetry^4^. In clinical routine, the vitality of the pulp, for example following traumatic dental injury or pulpitis, is assessed indirectly in simple subjective response to thermal or electrical stimulation. This approach allows for rapid evaluation; however, it is prone to errors due to its inherent subjectivity. Besides, the results from thermal and electrical tests of teeth represent the neural response of the pulp tissue to external stimuli and they are consequently only sensibility tests. Therefore, these tests do not allow reliable conclusions regarding the blood flow within the pulp tissue. Particularly, after a dental trauma, alteration of the pain threshold, changes in the supporting dental tissues or even a transient paresthesia of the pulp tissue may occur for several months^5^. This diagnostic dilemma leads to a considerable number of false-positive and false-negative test results, ultimately leading to over- or undertreatment^3,6^.

It is thus evident that a novel methodology for a direct and objective assessment of pulp vitality within a clinical context is essential for the advancement of diagnostic techniques. Dental magnetic resonance imaging has demonstrated its potential for the diagnosis in a broad spectrum of dental diseases, as evidenced by previous studies on caries lesions^7^, periapical lesions^8^, autotransplantation^9^, ectopic teeth^10^, and in pulp contrast enhancement analysis^11,12^. Here, only the comparisons of T1 sequences acquired before and after contrast agent administration have been able to demonstrate differences between healthy, well perfused teeth and teeth with pulp necrosis^11^. However, this approach has the disadvantage of requiring a contrast agent and the acquisition of two sequences instead of one to address the clinical question. Both limitations are clinically relevant. Consequently, novel contrasts like diffusion-weighted imaging (DWI) are a promising technique as they could address both shortcomings and thereby significantly enhance diagnostic capabilities. An ex vivo study has already demonstrated the potential for directly measuring the pulp’s diffusion^13^. However, these results cannot be directly translated to in vivo examinations, as an acquisition time of approximately 90 min is not feasible in a clinical setting and the diffusivity of extracted teeth is not comparable with in vivo measurements. Thus, no in vivo data of the pulp’s diffusive behavior is available.

In order to address the limitations of dental diffusion imaging, the turbo gradient spin echo (TGSE) BLADE sequence was selected for its capacity to reduce susceptibility artifacts by combining spin and gradient echoes, in contrast to echo planar imaging (EPI)-based diffusion sequences. Oversampling the k-space center using the underlying BLADE sequence also facilitates phase correction, which in turn reduces susceptibility to motion artifacts compared to a standard turbo spin echo sequence with Cartesian sampling^14^. This seemed necessary as the presence of susceptibility artifacts in the oral cavity, arising from the frequent bone-air and bone-soft tissue interfaces with its strong susceptibility differences as well as tooth restoration and replacement material, represents a significant challenge in dental MRI in general and particularly regarding DWI. In addition, tiny anatomical structures need to be resolved, resulting in substantial challenges for spatial resolution and consequently acquisition time. This issue was resolved by enhancing the time efficiency of TSE sequences through the incorporation of gradient echo (GRE) techniques. Therefore, the aim of this study was to assess the feasibility of the TGSE BLADE sequence at 3 T for high-resolution DWI of healthy dental pulps in vivo, to calculate reference values for the apparent diffusion coefficient (ADC) in healthy pulps, and to evaluate the influence of potential confounders (i.e. jaw and tooth type) on the ADC.

## Materials and methods

### Ethics, study participants and clinical vitality testing

This study was approved by the Ethics Committee of the University of Heidelberg (approval number S-452/2010). Informed written consent was obtained from all participants prior to the examinations. The study was designed and reported in accordance with the STROBE statement^15^ and has been performed in accordance with the Declaration of Helsinki.

Eligible volunteers underwent a comprehensive dental examination prior to MRI, which included sensibility testing of all teeth twice using refrigerant spray (Miracold^®^ Plus, −50 °C, Hager & Werken, Duisburg, Germany) applied via a cotton carrier. A positive (vital) sensibility test was defined as a reproducible response to cold stimulation on two separate occasions. Furthermore, only candidates without extensive carious lesions (i.e. no distinct cavities with visible dentine) and without clinical signs of pulpitis (positive sensitivity testing, positive percussion testing, visible signs of inflammation)^16^ were included, thereby excluding non-vital or partially vital teeth from further analysis. MR-based exclusion criteria at the tooth level included a history of root canal treatment, the presence of tooth crowns and of metal artifacts in the pulp region, and motion related artifacts. In total, 18 healthy volunteers (14 males, 4 females; mean age ± SD: 25.8 ± 6.5 years) were included.

### MRI image acquisition

All MRI measurements were performed on a 3 T MRI system (MAGNETOM PRISMA, Siemens Healthineers, Erlangen, Germany), using a dedicated 15-channel dental RF coil array (Mandibula Coil, Noras MRI Products GmbH, Hoechberg, Germany) to improve the signal-to-noise ratio in the dental region.

**Fig. 1:**
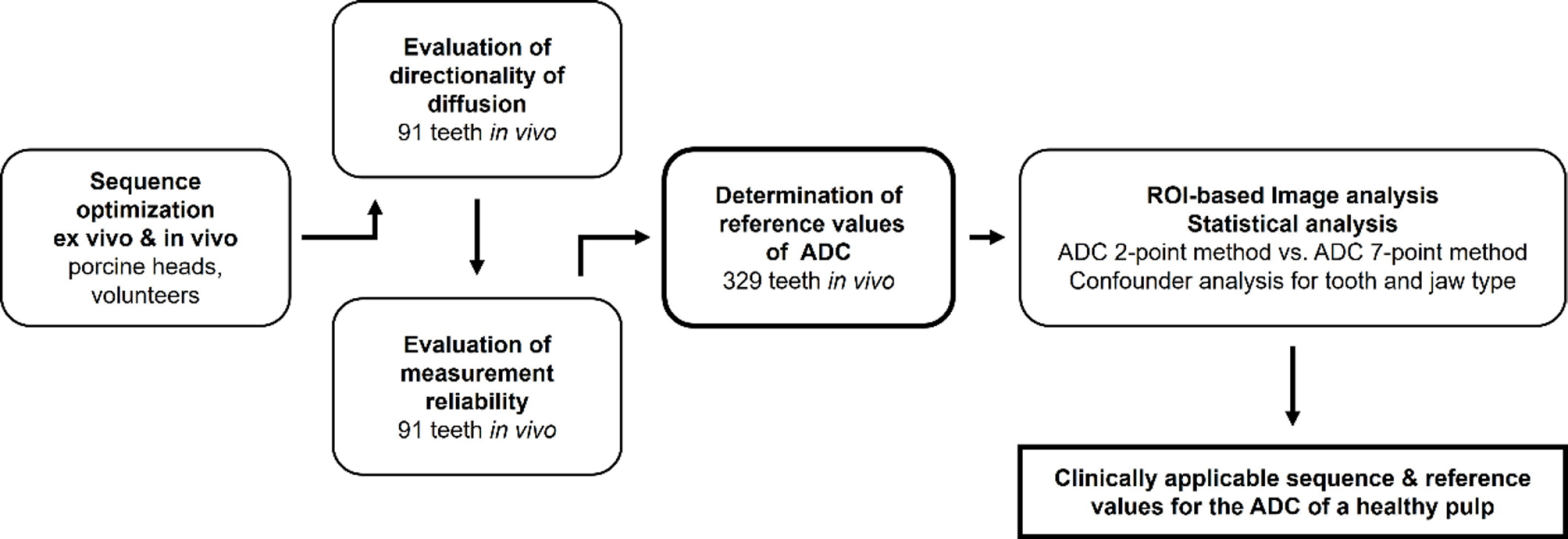
Study Workflow.

A turbo gradient spin echo BLADE research sequence was employed^14^. An overview of the study workflow consisting of optimization and analysis is depicted in Fig. 1. During the comprehensive sequence optimization process (both ex vivo and in vivo) special attention was given to the directionality of diffusion, the optimal b-value range, highest possible resolution within clinically applicable acquisition times, and intraindividual fluctuations. The orientation of neurovascular structures within the tooth relative to the MRI’s z-axis prompted the question whether there is a predominant direction of diffusion in the pulp. To investigate this, measurements on three subjects using a 7-point ADC were performed in each of the three gradient directions (acquisition time: 75:24 min). Regarding the optimal b-value range, the maximum suitable b-value was determined specifically to the tooth type, ensuring that the pulp signal displayed a clearly visible pulp-dentin border and was minimally affected by noise. Finally, intraindividual fluctuations of the ADC were assessed in three additional subjects performing three independent scans and comparing the calculated 7-point ADC (total acquisition time: 75:24 min).

In all 18 volunteers, the TGSE BLADE sequence parameters were the following: voxel size: 0.56 × 0.56 × 2.0 mm^3^; matrix size: 320 × 320; field of view (FOV): 180 × 180 mm^2^; number of slices: 16; repetition time: 2900 ms; echo time: 73 ms; flip angle: 150°; BLADE coverage: 241.3%; diffusion gradient direction: slice encoding direction; b-values: 0, 50, 200, 300, 400, 500, 600 s/mm^2^; number of averages: 2 (for each b-value); acceleration: simultaneous multi-slice (SMS) (factor 2); acquisition time (AT): 25:08 min.

### Image analysis

All MR images were analyzed by a physicist (B.Sc., with two years experience in dental MRI under supervision of a neuroradiologist with over ten years of experience in dental MRI) using the OsiriX Lite DICOM Viewer (version 13.0.2). For each subject, a region of interest (ROI) was manually drawn along the visible pulp-dentin border covering the pulp region on a slice of the b_0_ scan of the TGSE BLADE sequence for each maxillary and mandibular tooth. Subsequently, each ROI was transferred to the images of the remaining b-values. To correct for slight movement during image acquisition, ROIs were coregistered manually. For each ROI, the mean and standard deviation were then automatically calculated, with statistical analysis performed over all included voxels. Subsequently, two ADC values were calculated on a tooth type specific level: one using a 7-point approach (b_0_ to b_600_) to obtain the most accurate results, and another using a clinically feasible 2-point setup (b_0_ and b_600_)^17^. For the 7-point method, the ADC and its standard deviation was derived by fitting the appropriate function to the measured data.

### Statistical analysis

Data analysis was done using Python (version 3) with the help of Pandas (version 1.3.4) statistical functions and Scipy (version 1.7.1) using the curve fit function based on the method of least squares minimization.

Statistical analysis was performed using GraphPad Prism (version 7.0). Shapiro-Wilk test was used to test normal data distribution. The diffusion directions were analyzed using the Kruskal-Wallis test and intraindividual fluctuations were determined by calculating the mean of the difference between the ADC in the individual scans. Comparison between the two ADC calculation methods was done using the nonparametric Wilcoxon signed-rank test and the parametric one-way analysis of variance (ANOVA) was used for multiple comparisons of the eight tooth types. Statistical significance was defined as an alpha level of 5% (*p* < 0.05).

## Results

### Directionality of diffusion and reliability

**Fig. 2:**
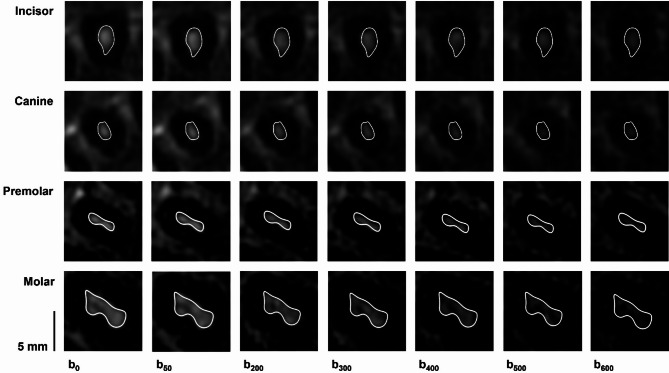
Example of the signal decay and region of interest positioning for increasing b-values in an incisor (first row), a canine (second row), a premolar (third row) and a molar (fourth row) of the maxilla.

The analysis of a total of 91 teeth from three subjects revealed significant differences when comparing diffusivity in phase-encoding (right-left) vs. frequency-encoding (anterior-posterior; *p* = 0.0116) and vs. slice-encoding direction (cranio-caudal; *p* = 0.0002). Diffusion in cranio-caudal direction (ADC_slice_ = 1.3549 ± 0.2586 × 10^−9^ m^2^/s) was significantly higher than in the transverse directions (ADC_frequency_ = 1.3130 ± 0.2397 × 10^−9^ m^2^/s; ADC_phase_ = 1.1958 ± 0.2726 × 10^−9^ m^2^/s). To minimize acquisition time in subsequent measurements, the diffusion gradient direction was set to slice-encoding direction. The maximum suitable b-value providing a pulp signal and not being influenced by noise was determined on a tooth-type specific level. The optimal b-value range was found to be 0, 50, 200, 400, 500, 600 (Fig. 2). Mean intraindividual ADC difference calculated from three scans in three subjects (91 teeth) were 0.178/0.137/0.163 × 10^−9^ m^2^/s (mean: 0.159 × 10^−9^ m^2^/s) for subjects 1/2/3.

### Reference values of ADC in healthy volunteers

In 18 healthy subjects, a total of 523 teeth were scanned. Of these, 194 were excluded from further analysis due to patient motion (101 teeth), avital suspected pulps identified in sensibility testing (4 teeth) and not clearly distinguishable pulp due to e.g. fillings or pulp stones (89 teeth). ADC analysis of the remaining 329 teeth yielded a mean value and standard deviation of 1.219 ± 0.340 × 10^−9^ m^2^/s using the 2-point method, while the 7-point method resulted in a slightly higher but otherwise comparable value of 1.242 ± 0.297 × 10^−9^ m^2^/s. The two methods showed a statistically significant difference (*p* ≤ 0.0001).

**Fig. 3:**
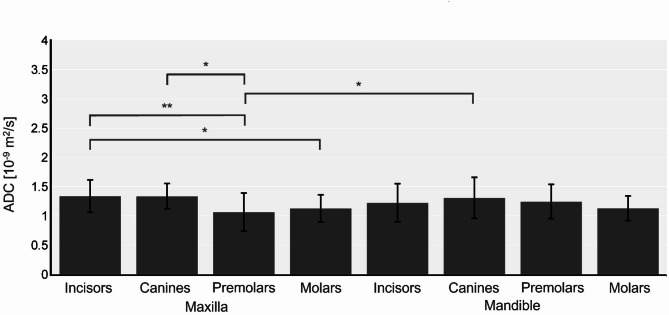
ADC considering b_0_ and b_600_ for all tooth types. Error bars represent the standard deviation of the mean. Significance: * (*p* ≤ 0.05), ** (*p* ≤ 0.01).

The tooth type specific subgroup analysis was performed using the 2-point method, as this resembles the clinical applicable method (Fig. 3; Table 1). It revealed significant differences between maxillary premolars ((1.065 ± 0.344) × 10^−9^ m^2^/s) vs. (1) maxillary incisors ((1.337 ± 0.340) × 10^−9^ m^2^/s; *p* = 0.0017), (2) maxillary canines ((1.335 ± 0.234) × 10^−9^ m^2^/s; *p* = 0.0207) and (3) mandibular canines ((1.308 ± 0.344) × 10^−9^ m^2^/s; *p* = 0.0473)); and maxillary incisory vs. maxillary molars ((1.128 ± 0.231) × 10^−9^ m^2^/s; *p* = 0.0401)).

**Table 1:** Reference values for the ADC in all tooth types. ADC: apparent diffusion coefficient.

Tooth Type	ADC (b_0_ to b_600_) [×10^−9^ m^2^/s]	ADC (b_0_, b_600_) [×10^−9^ m^2^/s]
Maxillary Incisors	1.315 ± 0.277	1.337 ± 0.340
Maxillary Canines	1.353 ± 0.217	1.335 ± 0.234
Maxillary Premolars	1.114 ± 0.325	1.065 ± 0.344
Maxillary Molars	1.175 ± 0.232	1.128 ± 0.231
Mandibular Incisors	1.181 ± 0.304	1.223 ± 0.423
Mandibular Canines	1.288 ± 0.350	1.308 ± 0.344
Mandibular Premolars	1.306 ± 0.293	1.244 ± 0.358
Mandibular Molars	1.158 ± 0.211	1.130 ± 0.186

## Discussion

This study demonstrates the feasibility and reliability of in vivo diffusion-based imaging of the human dental pulp using an optimized high-resolution TGSE BLADE sequence for the first time. Moreover, it establishes the optimal b-values and normal ADC values for the dental pulp as a basis for further studies. Furthermore, ADC values did not differ between tooth types, indicating that the ADC of the dental pulp is a stable marker that allows intra-individual comparison of different tooth types or different jaws. Thus, in vivo DWI holds promise for direct detection of pulp necrosis — a significant advance given that pulp vitality has traditionally been assessed indirectly (e.g. via electrical or thermal stimulation) with limited accuracy and reliability^3,4^. Currently, direct evaluation requires invasive pulp chamber access, typically reserved for ambiguous cases. As pathological alterations of the dental pulp are very frequent as consequence of various dental and periodontal diseases as well as traumatic dental injuries, the availability of a non-invasive, objective method assessing the dental pulp condition would improve diagnosis and treatment of a relevant patient cohort.

This study offers several strengths. First, a dedicated dental coil system was used enabling optimal coil design for superior signal-to-noise ratio compared to standard head-neck coils allowing together with the use of the novel hybrid sequence to reduce acquisition time from previously 90 min to 7 min^13,18^. Second, two approaches were applied to calculate the ADC, thereby enhancing the robustness of the data and allowing a comprehensive evaluation of the accuracy of ADC calculations based on two b-values only, as used in clinical examination. Despite the small sample size (18 subjects), over 300 teeth and 2300 ROIs were analyzed, and potential confounders (tooth and jaw types) were systematically evaluated.

To authors’ knowledge, this is the first in vivo study analyzing DWI parameters in the human pulp. One ex vivo study reported a slightly lower ADC values for previously vital teeth (1.05 ± 0.10) × 10^−9^ m^2^/s^13^ compared to the in vivo results in this study ((1.219 ± 0.340) × 10^−9^ m^2^/s). Another ex vivo study reported a large ADC range of healthy pulps of 0.5–1.7 × 10^−9^ m^2^/s but with a mean of 1.24 ± 0.06 × 10^−9^ m^2^/s, which is very similar with our in vivo results^19^. This deviation can be attributed to the interrupted vascular supply after tooth extraction and the tissue fixation processes being used (e.g. formalin). Transferability of the ex vivo results is, consequently, limited and in vivo measurements are essential for the definition of normal values. Nevertheless, the ex vivo results are important as they highlight the potential of DWI-based pulp assessments by illustrating a significant difference between intact and decayed pulp tissue (1.0 vs. 0.74–0.89 × 10^−9^ m^2^/s and 0.5–1.7 vs. 0.3–1.0 × 10^−9^ m^2^/s) with histological agreement^13,19^. Given the non-overlapping mean ADC and its standard deviation in our study (1.219 ± 0.340 × 10^−9^ m^2^/s) and the previously reported ADC deviations for pathologies it seems likely that this technique can differentiate between healthy and necrotic pulps in vivo.

Although the reported difference between intact and decayed teeth was 0.11–0.26 × 10^−9^ m^2^/s^13^, the maximal mean ADC standard deviation per tooth type in this study was relatively high (0.423 × 10^−3^ mm^2^/s for maxillary incisors), potentially limiting clinical applicability. Substantially lower SDs were observed (e.g., 0.186 × 10^−9^ m^2^/s for mandibular molars). This variability is likely due to difference in pulp volume (smallest pulp volume present in mandibular incisors, and largest in molars) and consequently presents a limitation due to partial volume effects (in-plane and even more through-plane). Next, the intraindividual fluctuations analyzed in several subjects (mean: 0.159 × 10^−9^ m^2^/s) were even lower than the minimal mean ADC standard deviation per tooth type. A comparison of these results with standard ADC values from in vivo studies of other tissues revealed comparable results in reactive cervical lymph nodes (1.293 ± 0.32 × 10^−9^ m^2^/s; our data 1.219 ± 0.34 × 10^−9^ m^2^/s)^20^. Standard deviation for mean results (± 0.34 × 10^−3^ mm^2^/s) as well as intraindividual retest stability (0.159 × 10^−9^ m^2^/s) were comparable to standard deviation noted for cervical lymph node assessment (0.32 × 10^−9^ m^2^/s)^20^.

The maximal mean ADC standard deviation per tooth type was larger than the differences between the potential confounders jaw- (maximal difference: 0.179 × 10^−9^ m^2^/s) and tooth type (maximal difference: 0.272 × 10^−9^ m^2^/s). This implies that intraindividual ADC values of different tooth types can be directly compared and that there may not be a need of cross-platform reference ADC values, which are associated with several difficulties^21^. Comparable results were previously reported for contrast-enhanced MR examinations of the pulp^12^. In a comprehensive study of 50 age- and sex-controlled volunteers analysis of 1585 teeth revealed also significant but only minor differences between tooth- and jaw type as in this study^12^. For illustrative purposes, the ADC values of the healthy pulp are observed to fall within the range of benign breast lesions (1.19 to 1.73 ± 0.34) × 10^−9^ m^2^/s^22^. The reference ADC for grey matter is found to be significantly lower ((0.89 ± 0.04) × 10^−9^ m^2^/s)^23^.

Several limitations must be acknowledged. First, this study focused on healthy volunteers, leaving diagnostic challenges related to dental restorations and replacement materials unaddressed. Second, despite reducing acquisition time from 90 min as described in other studies^13^ to 7 min in our study, there still have been relevant dropouts on tooth level due to motion artifacts. In addition, morphology and eruption direction of wisdom teeth may substantially differ compared to other molars. Within this study cohort only 12 wisdom teeth were analyzed and therefore no additional subgroup analysis was performed. A detailed investigation of anatomical variants would be an important and valuable next step in future studies with larger cohorts. The absence of an inter- and intra-observer agreement assessment in the ROI placement and ADC measurement process limits the study. Future studies should address this by evaluating inter- and intra-observer reliability to further validate the robustness and reproducibility of the methodology. Moreover, in teeth with small pulps like mandibular incisors, we observed partial volume effects. The use of identical numbers of signal averages for both b = 0 and b = 600 images may have contributed to a reduced signal-to-noise ratio at higher b-values and should be considered in the future. Further improvements in scanning efficiency, potentially through AI-based postprocessing^24–26^, are needed to enhance clinical applicability in terms of even higher structural resolution as well as lower acquisition times.

## Conclusion

This prospective in vivo study established and validated an optimized method for characterizing and quantifying the diffusion properties of the dental pulp for the first time. Reliable reference ADC values were obtained, and no relevant difference between jaw- or tooth types were observed, indicating that the dental pulp ADC is a stable intra- and inter-individual marker. These findings may substantially improve diagnostic workup of various pulp diseases and help minimize over- and undertreatment in the future.

## Data Availability

The datasets used and/or analysed during the current study are available from the corresponding author on reasonable request.
